# FoCupin1, a Cupin_1 domain-containing protein, is necessary for the virulence of *Fusarium oxysporum* f. sp. *cubense* tropical race 4

**DOI:** 10.3389/fmicb.2022.1001540

**Published:** 2022-08-30

**Authors:** Tiantian Yan, Xiaofan Zhou, Jieling Li, Guanjun Li, Yali Zhao, Haojie Wang, Huaping Li, Yanfang Nie, Yunfeng Li

**Affiliations:** ^1^College of Materials and Energy, South China Agricultural University, Guangzhou, China; ^2^Guangdong Province Key Laboratory of Microbial Signals and Disease Control, College of Plant Protection, South China Agricultural University, Guangzhou, China

**Keywords:** *Fusariumoxysporum* f. sp. *cubense* tropical race 4, FoCupin1, effector, virulence, banana Fusarium wilt

## Abstract

*Fusarium oxysporum* f. sp. *cubense* tropical race 4 (Foc TR4) is an important soilborne fungal pathogen that causes the most devastating banana disease. Effectors secreted by microbes contribute to pathogen virulence on host plants in plant-microbe interactions. However, functions of Foc TR4 effectors remain largely unexplored. In this study, we characterized a novel cupin_1 domain-containing protein (FoCupin1) from Foc TR4. Sequence analysis indicated that the homologous proteins of FoCupin1 in phytopathogenic fungi were evolutionarily conserved. Furthermore, FoCupin1 could suppress BAX-mediated cell death and significantly downregulate the expression of defense-related genes in tobacco by using the *Agrobacterium*-mediated transient expression system. *FoCupin1* was highly induced in the early stage of Foc TR4 infection. The deletion of *FoCupin1* gene did not affect Foc TR4 growth and conidiation. However, *FoCupin1* deletion significantly reduced Foc TR4 virulence on banana plants, which was further confirmed by biomass assay. The expression of the defense-related genes in banana was significantly induced after inoculation with *FoCupin1* mutants. These results collectively indicate *FoCupin1* is a putative effector protein that plays an essential role in Foc TR4 pathogenicity. These findings suggest a novel role for cupin_1 domain-containing proteins and deepen our understanding of effector-mediated Foc TR4 pathogenesis.

## Introduction

Banana (*Musa* spp.) is one of the most important fruits worldwide, which provides vital nutrition to millions of people (Ordonez et al., 2015). Banana Fusarium wilt, caused by *Fusarium oxysporum* f. sp. *cubense* (Foc), is a soil-borne fungal epidemic disease with rapid onset and severe damage, causing serious economic losses to the global banana industry (Orr and Nelson, 2018; An et al., 2019). According to the pathogenicity of different banana strains, Foc is classified into three races, including Foc1, Foc2, and Foc4 (Guo et al., 2015; Dong et al., 2020). Foc4 has been further divided into tropical race 4 (Foc TR4) and subtropical race 4 (Foc STR4), which affect the Cavendish bananas in the tropics and subtropics, respectively (Ploetz, 2015). Among Foc races, Foc TR4 has the strongest pathogenicity, and spread from the Asia-Pacific region to the Middle East, Africa, Australia, and Latin America (Thangavelu et al., 2021; Zhang et al., 2021). Despite the serious damage caused by Foc TR4, efficient strategies for its management are not available to date and the understanding of its pathogenesis is still rudimentary (Liu et al., 2019).

Pathogenic fungi infect host plants by complex mechanisms, generally by secreting a wide range of pathogenic factors to evade or suppress their host defenses and cause disease (Pradhan et al., 2021). Among these, effectors are key virulence determinants of pathogenic fungi and play crucial role in successful pathogenesis, predominantly by avoiding the host-surveillance system (Chang et al., 2019; Vincent et al., 2019). In Foc-banana interactions, effectors have been proven to act as pathogenicity factors and play important roles during the early infection stages (Czislowski et al., 2018). Previous studies have reported several effectors in *Fusarium oxysporum*, including secreted in xylem (SIX; Czislowski et al., 2018), Necrosis proteins (NPP1; Oome et al., 2014), Cerato-Platanin (Liu et al., 2019), hydrophobins (Casarrubia et al., 2018), and M35 family metalloproteinases (Zhang et al., 2021), which are all required by Foc4 for its penetration and full virulence.

Cupins are a superfamily of topologically conserved but functionally diversified proteins that exist in all kingdoms of life (Khuri et al., 2001). The reported functions of cupins include isomerases, epimerase, dioxygenase, and nonenzymatic storage proteins (Uberto and Moomaw, 2013). Xu et al. (2010) reported that TaARD, a novel aci-reductone-dioxygenase in the cupin superfamily, was involved in wheat defense response and ethylene signaling in response to biotic and abiotic stresses (Xu et al., 2010). Germin-like proteins (GLps) are a diverse and ubiquitous family of plant glycoproteins belonging to the cupin superfamily. A GLP gene *GhABP19* was isolated from *Gossypium hirsutum* and was found to play important roles in the regulation of plant resistance to *Verticillium* and Fusarium wilt (Pei et al., 2019). Another GLP2 protein from *Gossypium hirsutum* was also proved to have important functions in plant defense responses against *V. dahliae*, *F. oxysporum,* and in responses to oxidative stress (Pei et al., 2020). Furthermore, cupin proteins have also been identified in some phytopathogens with different functions in virulence. The VdQase protein from *Verticillium dahliae*, which has a conserved functional domain of cupin, was predicted to have a quercetinase activity. The *VdQase* deletion mutants of *V. dahliae* exhibited reduced pathogenicity and *VdQase* was proved to be a key factor for *V. dahliae* in countering potato defenses (Abdelbasset et al., 2015). SsYCP1, a YML079-like cupin protein from *Sclerotinia sclerotiorum*, was characterized as a putative effector protein contributing to fungal pathogenicity (Fan et al., 2021). Although these reports indicate that cupin proteins are involved in some fungal pathogenicity, the underlying mechanisms remain to be revealed.

In our previous work, a shotgun-based proteomic approach was employed to identify the secreted proteins of Foc TR4, followed by high-quality secretome prediction and in-depth *in silico* analysis, which provided a resource for studying Foc TR4 candidate effectors (He et al., 2021). Here, we analyzed a candidate effector, a Cupin type-1 domain-containing protein (named as FoCupin1), using the *Agrobacterium*-mediated transient expression system in tobacco. The expression of FoCupin1 was significantly induced in the early stage of Foc TR4-banana interaction, as determined *via* the RT-qPCR method. The FoCupin1 protein possessed a signal peptide, had no transmembrane domain or GPI-anchor site, and was predicted to have an extracellular localization. Sequence analysis demonstrated that the homologs of FoCupin1 in phytopathogenic fungi were evolutionarily conserved. Analyses of the *FoCupin1* knockout mutant indicated that FoCupin1 is required for the pathogenicity of Foc TR4 and suppressed the defense responses in banana plantlets, while it did not affect fungal growth and conidiation. Our results suggest that FoCupin1 might be an important pathogenic factor and play a virulence role in the Foc TR4-banana interaction.

## Materials and methods

### Plant, fungal strains, and growth conditions

The Foc TR4 strain DZ1 was used as the wild-type control in this study, which was confirmed the pathogenicity by inoculation onto the host banana cultivars in our previous study (Qin et al., 2017). The *FoCupin1* gene deletion mutants were derived from this isolate. The wild-type strain and the transformants generated in this study were grown at 28°C on PDA medium for 5 days to assess growth and colony characteristics. Czapek Dox (CD) medium was used for conidiation assays and liquid NCMB medium was used to mimic the Foc-banana interaction as described (He et al., 2021). The Foc TR4-susceptible banana cultivar Brazilian (AAA group, Cavendish) and tobacco (*Nicotiana benthamiana*) were used in this study. Plants were grown in a greenhouse at 25°C ± 1°C, 70%–80% relative humidity with a 12-h photoperiod (250 μmol m^−2^ s^−1^). Banana plantlets at fully fourth-leaf stage and five-week tobacco plants were used for further experiments. The fungal conidia were diluted to approximately 1 × 10^5^ conidia/mL for further use.

### Bioinformatic analysis

Five internet-based tools, SignalP 4.0, WoLF PSORT, TargetP 1.1, TMHMM Server 2.0, and big-PI predictor, were employed to analyze FoCupin1 as described (He et al., 2021). The candidate effector analysis was predicted using the online predictor tool EffectorP 2.0^1^ with setting the class probability threshold to 0.5 (Sperschneider et al., 2018). The domains of FoCupin1 were identified by Pfam^2^ (Mistry et al., 2021). Homology searches were performed using BLAST tools.^3^ Phylogenetic trees were constructed using the maximum-likelihood method in the MEGA X software package with a bootstrap test with 1,000 replicates (Kumar et al., 2018).

### Gene deletion and complementation

The △*FoCupin1* mutant strain was constructed by homologous recombination as previously described (Liu et al., 2019). Briefly, the 5′- and 3′-flanking sequences of the *FoCupin1* gene were amplified using the genomic DNA of DZ1 strain as a template with the ExTaq polymerase (TaKaRa, China). The *FoCupin1* gene was replaced by a hygromycin-resistance cassette (*hph*), which was driven by a constitutive TrpC promoter amplified from the pCT74 vector. PEG-mediated protoplast transformation was adopted in this study. The putative deletion mutants were identified by PCR analysis and further confirmed by southern blot. To further verify the function of *FoCupin1*, gene complementation was conducted by transformation of *FoCupin1*-deleted mutant with the complementation vector pCTZN (zeocin resistance), which was constructed by introducing the entire coding region of *FoCupin1* with its native promoter and terminator. The protoplasts released from the *FoCupin1*-deleted strain were transformed and putative complementation were selected with zeocin and further examined *via* PCR with primer pair *FoCupin1*-comF/R (Supplementary Table S1). Primers for PCR and probe construction used in this study were listed in Supplementary Table S1.

### Stress sensitivity, hyphae dry weight determination, and cellophane membrane assays

To determine the difference in stress responses between WT and mutants, strains were cultured on PDA plates supplemented with the final concentrations of 1 mol/l NaCl, 1 mol/l M Sorbitol, 0.05% w/v SDS, 200 μg/ml congo red (CR), 100 μg/ml calcofluor white (CFW) or 300 mM H_2_O_2_ for 5 days at 28°C. In order to determine whether there is any phenotypic difference between WT, △*FoCupin1*, and △*FoCupin1*-com, same spore solution concentration inoculated into CM medium (10 g of glucose, 2 g peptone, 1 g yeast extract, 1 g casamino acids, nitrate salts, trace elements, 0.01% of vitamins, 10 g agar and 1 l water, pH 6.5) for one or 3 days in the incubator at 28°C, 120 rpm. The experiments were repeated three times. Data were analyzed using the ANOVA procedure of SPSS.

### Reverse transcription quantitative PCR analysis

Total RNA was extracted from Foc TR4 using a Fungal RNA kit (Omega, United States) according to the manufacturer’s protocol as described previously (He et al., 2021). Briefly, banana roots were grounded thoroughly with liquid nitrogen. A dialysis bag (Sigma-Aldrich D0530, molecular weight cut-off of 12,400) enclosing 15 ml of NCM medium plus 15 ml of banana root extract was then placed into 250 ml of NCM medium. The mycelia were collected for RNA preparation. Total RNA from tobacco and banana was extracted using Plant RNA Kit (Omega, United States) following the manufacturer’s instructions. The Reverse transcription quantitative PCR (RT-qPCR) was performed on a CFX Coxnnect™ Real-Time System (Bio-Rad, Hercules, CA, United States) with the SYBR Premix Ex Taq Kit (TaKaRa, Beijing, China) according to the manufacturer’s instructions. For Foc TR4, the *FoEF1a* gene was used as reference. For banana and tobacco, *MaActin* and *NbEF1a* were used as internal controls, respectively. All gene-specific primers for RT-qPCR (Supplementary Table S1) were designed using Primer 5.0 software. Relative transcript levels for each gene were calculated using the 2^−△△CT^ method (Livak and Schmittgen, 2001).

### Transient expression of *FoCupin1* in *Nicotiana benthamiana*

The open reading frame of FoCupin1 with the N-terminal signal peptide (named as SPFoCupin1) and without the signal peptide (named as NSPFoCupin1) was amplified by PCR and inserted into the pBI121 vector. The recombinant constructs were transformed into *Agrobacterium tumefaciens* strain GV3101 through electroporation method and then transiently expressed in *N. benthamiana* leaves according to the methods of Ma et al. (2012). In the *A. tumefaciens*-mediated transient expression assays, Bcl2-associated X protein (BAX) and the translationally controlled tumor protein (TCTP) were used as positive and negative controls, respectively. Each treatment was performed on three leaves from six individual plants, and the assay was repeated at least three times. The inoculated leaves were photographed in 3–4 days after infiltration.

### Pathogenicity tests and fungal biomass evaluation

The inoculation method was performed as described previously (Dong et al., 2019). Banana plantlets roots were soaked in freshly prepared spores suspension (1 × 10^5^ conidia/ml) for 30 min and then were planted back to the pots. Inoculated plants were kept in a humidity chamber at 25°C ± 1°C. Disease symptoms were recorded 28 days after inoculation and disease indices were calculated according to the methods described previously (An et al., 2019). The experiment was repeated three times and each treatment used 30 banana plantlets. Cellophane penetration assay was performed as previously described (Dai et al., 2016). For the fungal biomass assay, a small piece of infected banana root tissue (1 cm) was cut for DNA extraction using a Fungal DNA kit (Omega, United States) according to the manufacturer’s protocol. DNA-based qPCR was performed using a CFX Coxnnect™ Real-Time System (Bio-Rad, Hercules, CA, United States). Relative fungal biomass was calculated as a ratio (*FoEF1α*/ *MaActin*) represented by the equation 2^[CT(*MaActin*)-CT(*FoEF1a*)]^ as previously described (Park et al., 2012).

### Analysis of fusaric acid

Fusaric acid (FA) assay was performed according the method as described with some modification (Dong et al., 2019). Briefly, fungal mycelium was cultured in CD medium on a rotary shaker with 140 rpm in continuous illumination (5,000 Lux) for 9 days at 30°C. The culture solution was ultrasonicated for 10 min (Ruisheng Instrument and Meter Co., Ltd., China), then filtered with double layers of gauze. The filtrate was centrifuged at 6,000 rpm for 30 min, and the supernatant was concentrated under reduced pressure at 45°C. After centrifugation at 5,000 rpm for 20 min, the supernatant was filtered through a sterile filter paper, and the filtrate was stored at 4°C. FA contents were detected by monitoring the absorbance at 268 nm with pure Fusaric acid (Sigma-Aldrich, St. Louis, MO, United States) as standard.

### Detection of reactive oxygen species

The tobacco leaves and banana roots were sampled after agroinfiltration or fungal inoculation. DAB (3,3′-diaminobenzidine) staining was used for *in situ* detection of H_2_O_2_ as previously described (Thordal-Christensen et al., 1997). H_2_O_2_ was measured using the titanium tetrachloride precipitation method (Brennan and Frenkel, 1977).

### Statistical analysis

Statistical analysis was performed using the SPSS 14.0 software. All values were presented as mean values ± standard deviations. Statistically significant differences were determined using Duncan’s multiple range tests (*p* < 0.05) or Student’s *t*-test (*p* < 0.05), respectively.

## Results

### *FoCupin1* is conserved among different *Fusarium* strains

**Figure 1:**
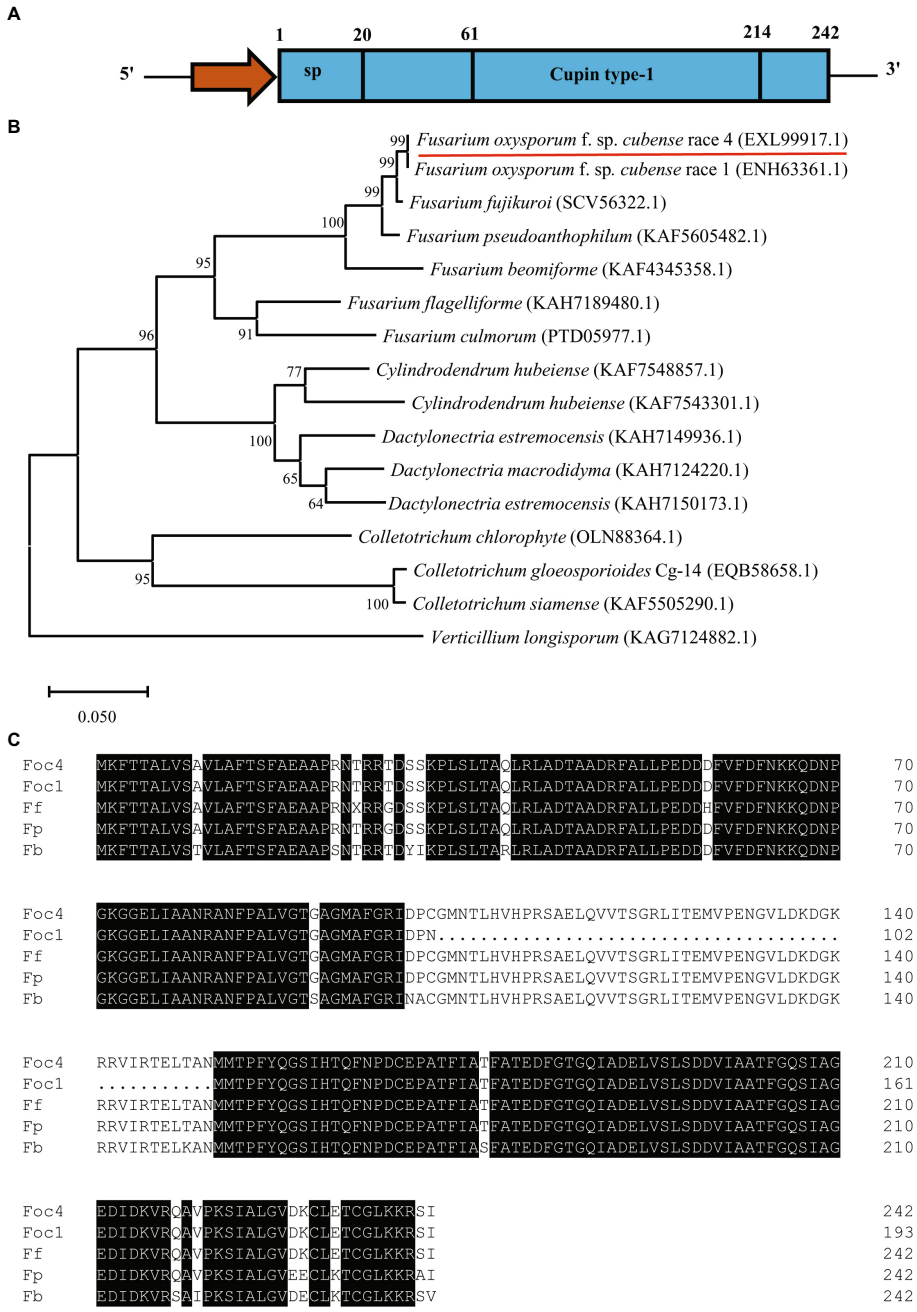
Bioinformatic analysis of FoCupin1. **(A)** Schematic structure of the FoCupin1 protein. The signal peptide (sp) and the domain were predicted by SignalP and Pfam, respectively. **(B)** Phylogenetic analysis of the FoCupin1 protein. Maximum likelihood tree was built from FoCupin1 and its orthologous proteins from 16 different fungal pathogens. **(C)** Amino acid sequence alignment of *FoCupin1* and its homologs in other species, including *Fusarium oxysporum* f. sp*. cubense* race 1 (ENH63361.1; Foc1), *F. fujikuroi* (SCV56322.1; Ff), *F. pseudoanthophilum* (KAF5605482.1; Fp)*, F. beomiforme* (KAF4345358.1; Fb). The shaded dark background indicates conserved common amino acids, and the dots represent gaps in the amino acid sequences.

The gene encodes a 242 amino acid protein with a Cupin_1 domain based on Pfam analysis (Figure 1A). Cupin1 in Foc TR4 (FoCupin1) was predicted to contain a signal peptide (by SignalP) but no transmembrane domain (by TMHMM2) or GPI anchoring signal (by Big-PI), and was predicted to be a extracellular protein (by WoLF PSORT and TargetP). Thus, FoCupin1 was classified as classically secreted protein. A BlastP search against the NCBI Non-Redundant protein database indicated that FoCupin1 shared a high degree of similarity with several proteins of plant fungal pathogens, including related to spherulin 1A precursor (SCV56322.1; similarity: 98.76%) from *F. fujikuroi*, spherulin 1A precursor (KAF5605482.1; similarity: 97.93%) from *F. pseudoanthophilum*, Spherulin-1B (ENH63361.1; similarity: 79.75%) from Foc1, hypothetical protein RmlC-like cupin domain-containing protein (KAH7124220.1; similarity: 79.34%) from *Dactylonectria macrodidyma*, hypothetical protein G7Z17_g6790 (KAF7548857.1; similarity: 78.93%) from *Cylindrodendrum hubeiense*, and Spherulin-1A 1 (OLN88364.1; similarity: 70.73%) from *Colletotrichum chlorophyti*, respectively (Figure 1B). Phylogenetic analysis also showed that the FoCupin1 and related to spherulin 1A precursor from *F. fujikuroi* are the closest, sharing 98.76% sequence identity at the protein level (Figure 1C).

### *FoCupin1* is highly upregulated during early infection process

**Figure 2:**
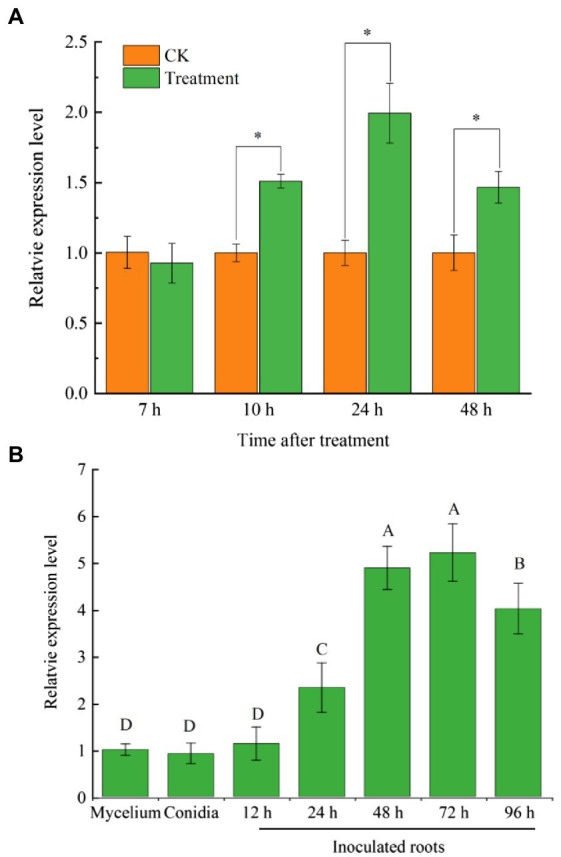
RT-qPCR analysis of *FoCupin1* expression. **(A)** *FoCupin1* expression in Foc TR4 conidia cultured in NCM medium plus banana plant extracts. **(B)** *FoCupin1* expression at different developmental and infective stages. The fungal constitutive gene *FoEF1α* was used as internal reference. Values are the means (±SE) based on three independent experiments and bars indicate standard deviations. Asterisks and different letters indicate statistical significance (*p* < 0.05) using Student’s *t*-test.

FoCupin1 was identified from the secretome of banana root extract-induced Foc TR4 mycelium. To investigate the expression patterns of *FoCupin1* in Foc TR4, RT-qPCR assays were firstly conducted using Foc TR4 conidia cultured in NCM medium plus banana root extracts at four time points. The expression of *FoCupin1* was significantly induced in Foc TR4 at 10, 24, and 48 h after inoculation of conidia, which peaked at 24 h (Figure 2A). To further investigate *FoCupin1* expression at different developmental and infection stages, RT-qPCR assays were conducted using samples from vegetative mycelium, conidia, and infected root after inoculation with Foc TR4 conidia on banana plantlets. The expression of *FoCupin1* was low in the vegetative mycelium and spore stages, and was significantly induced at 24 h after induction by banana extracts, which peaked at 72 h (Figure 2B). These results showed that *FoCupin1* was upregulated during infection, indicating that FoCupin1 may play an important role in Foc TR4-banana interaction.

### Expression of *FoCupin1* inhibits plant immune responses in *Nicotiana benthamiana*

**Figure 3:**
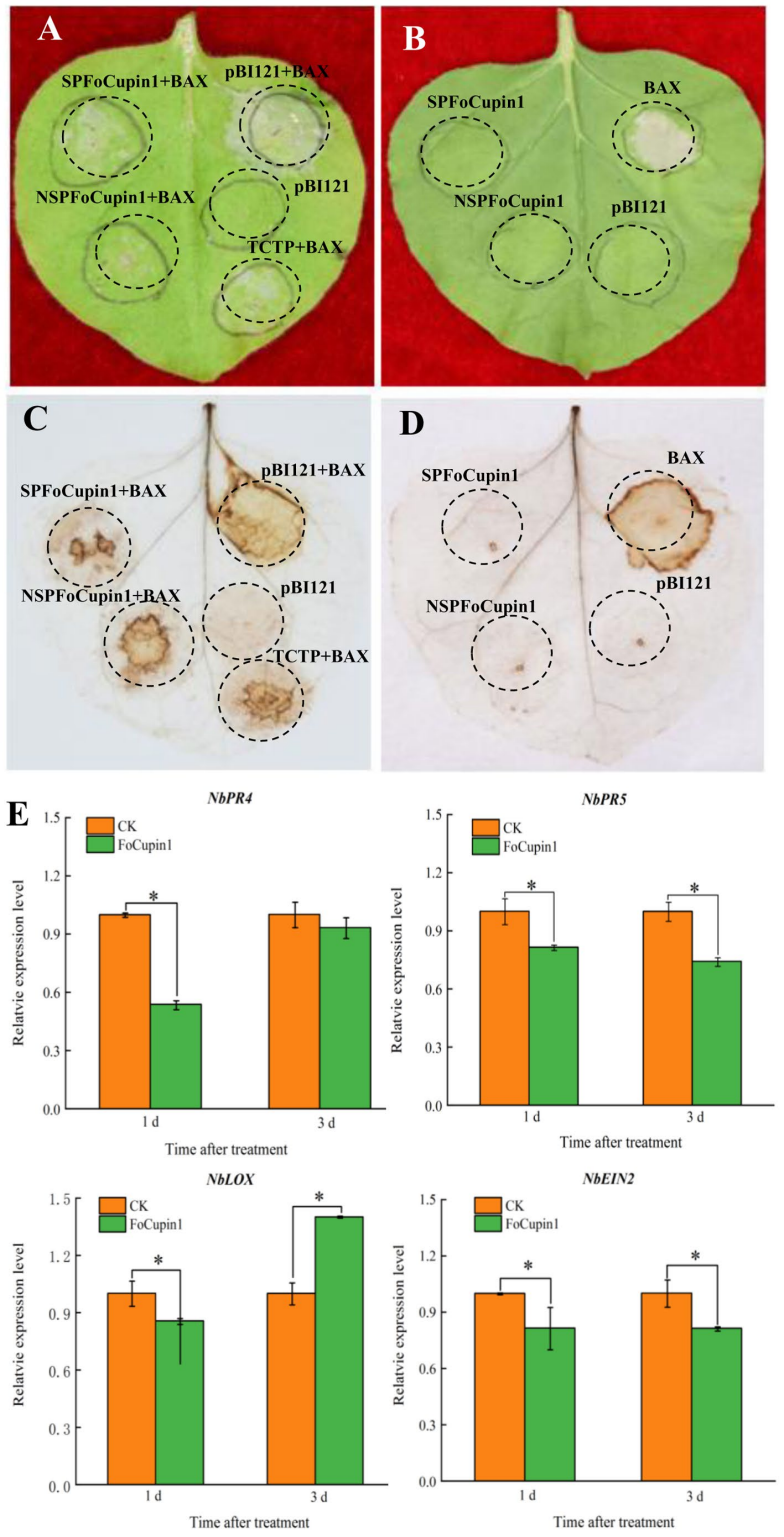
FoCupin1 suppresses plant immune responses in *Nicotiana benthamiana.* **(A)** FoCupin1 could suppress the BAX-mediated cell death in *N. benthamiana* leaves. Tobacco leaves were infiltrated with *Agrobacterium tumefaciens* expressing SPFoCupin1, NSPFoCupin1, TCTP (as positive control), or pBI121 empty vector (as negative control) 2 days before infiltration with *A. tumefaciens* expressing BAX. The cell death was photographed 3–4 days after infiltration. **(B)** FoCupin1 could not induce the cell death in *N. benthamiana* leaves. **(C)** Reactive oxygen species (ROS) accumulation in **(A)** were detected by DAB staining. **(D)** ROS accumulation in **(B)** were detected by DAB staining. **(E)** RT-qPCR analysis of four defense-related genes after infiltration with *A. tumefaciens* expressing FoCupin1. Values are the means (±SE) based on three independent experiments and bars indicate standard deviations. Asterisks indicate statistical significance (*p* < 0.05) using Student’s *t*-test.

To determine the immunosuppressive ability of FoCupin1, we constructed SPFoCupin1 and NSPFoCupin1 into the plant transient expression vector pBI121 plasmid, respectively. The agrobacteria culture containing constructs was injected into *N. benthamiana* leaves for transient expression using an *Agrobacterium*-mediated transformation system. The BAX protein, a member of the Bcl-2 family of mouse, has been shown to strongly induce cell death in *N. benthamiana* (Lacomme and Santa Cruz, 1999); TCTP, a protein well known in animals, has been characterized as a functional BAX suppressor protein to inhibit plant cell death (Hoepflinger et al., 2013). BAX and TCTP are used as negative and positive controls in this study, respectively. After 3 days of infiltration, both SPFoCupin1 and NSPFoCupin1 could suppress BAX-mediated cell death in *N. benthamiana* leaves (Figure 3A), while neither of them could induce the cell death (Figure 3B). DAB staining showed that both SPFoCupin1 and NSPFoCupin1 could inhibit ROS accumulation in *N. benthamiana* leaves, compared to BAX as a negative control and TCTP as a positive control (Figure 3C). However, SPFoCupin1 and NSPFoCupin1 could not induce ROS accumulation (Figure 3D). These results suggested that the transient expression of FoCupin1 could suppress the plant defense responses. Four defense-related marked genes, namely *NbPR5* and *NbPR4* for salicylic acid (SA), *NbLOX* for jasmonic acid (JA), and *NbEIN2* for ethylene-dependent immunity (Zhang et al., 2010; Zhang, 2020; Wang et al., 2021), were selected for expression analysis *via* RT-qPCR after infiltration by *FoCupin1* (Figure 3E). The expression of these genes decreased significantly in *N. benthamiana* leaves with transient expression of *FoCupin1*, further indicating that *FoCupin1* may suppress plant immune responses by inactivating the SA-, JA-, and/or ethylene-mediated defense pathways.

### *FoCupin1* deletion has no effect on fungal growth and development

**Figure 4:**
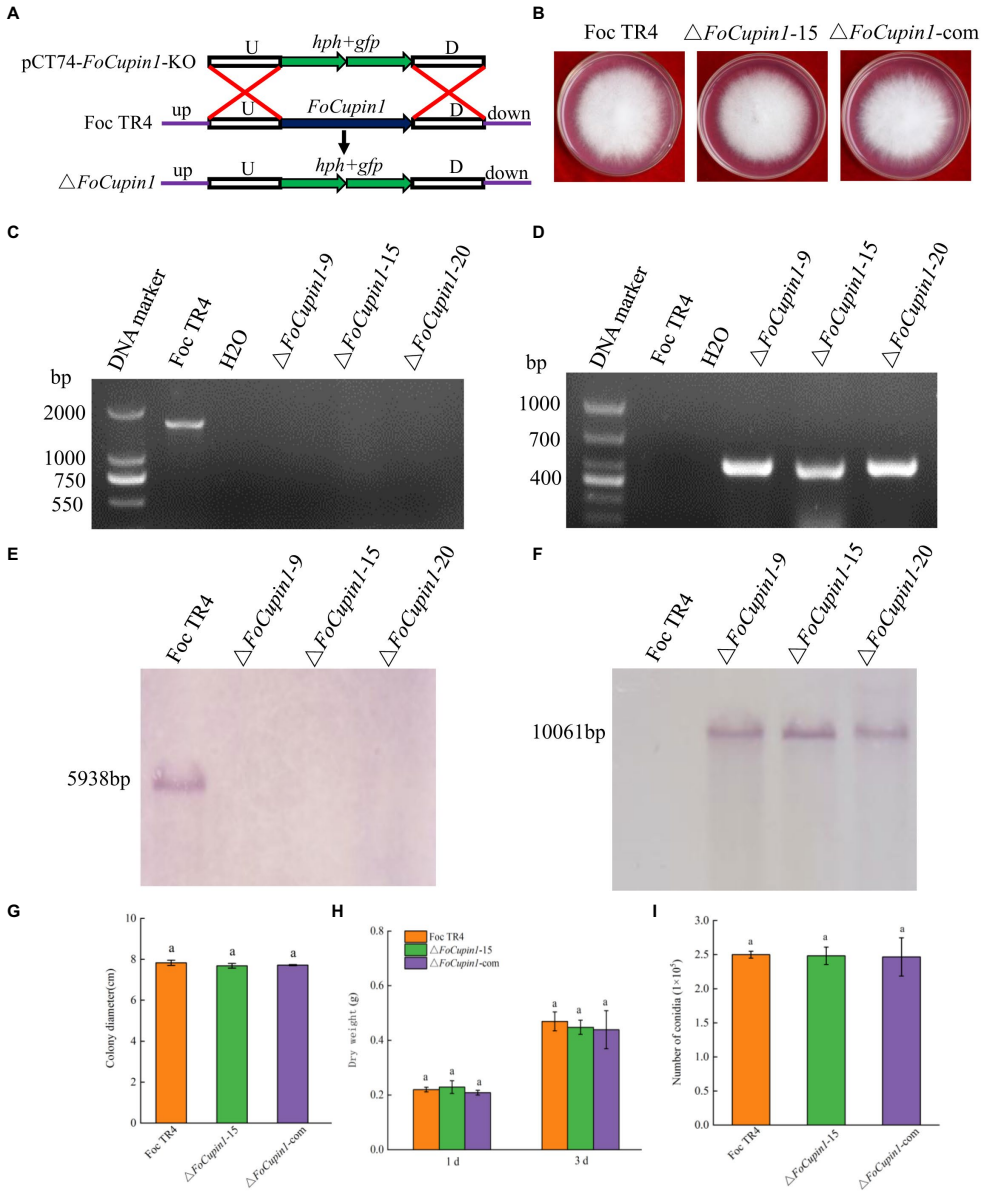
Construction, confirmation, and characterization of *FoCupin1* deletion and complementation mutants. **(A)** Schematic strategy used for generation of the deletion mutant according to homologous recombination. U, upstream flanking region of *FoCupin1*; D, downstream flanking region of *FoCupin1*. **(B)** Colony morphologies on PDA medium. Photos were taken 5 days after incubation. **(C,D)** PCR confirmation using *FoCupin1* and *hph* as probes; **(E,F)** Southern blot validation using *FoCupin1* and *hph* as probes, respectively. **(G)** Colony diameter. **(H)** Dry weight. Hyphae were collected from 1 and 3 days cultures grown in CM medium. **(I)** Conidiation. Foc TR4, the wide-type strain; *FoCupin1-9, FoCupin1-15* and *FoCupin1-20*, *FoCupin1* deletion mutants; △*FoCupin1*-com, *FoCupin1* complementation strain. Means and standard deviations were calculated from three independent experiments. Values on the bars followed by the same letter are not significantly different at *p* < 0.05 by Duncan’ s multiple range test.

To determine the function of the *FoCupin1* gene in Foc TR4, we generated a gene replacement construct containing a hygromycin-resistance gene cassette (*hph*) and transformed it into Foc TR4 (Figure 4A). Fourteen preliminary *FoCupin1* mutants were identified *via* PCR screening from 32 hygromycin-resistant transformants (Figures 4C,D). Three mutants (△*FoCupin1*-9, △*FoCupin1-*15, and △*FoCupin1-*20) were further verified by southern blot analysis using a *hph*-specific probe or *FoCupin1*-specific probe, the results of which showed that the mutants contained *hph* gene and lacked *FoCupin1* gene (Figures 4E,F). The expression of *FoCupin1* in the candidate deletion mutants was measured by RT-qPCR, the results of which confirmed that the mutants lacked *FoCupin1* (Supplementary Figure S1). No morphological differences were observed between the three deletion mutants on MM medium and CM medium (Supplementary Figure S2), thus △*FoCupin1*-15 was selected as a representative for further analyses. To further test if the effect was exclusively due to the deletion of *FoCupin1*, two complementation strains (△*FoCupin1*-9-com and △*FoCupin1-*15-com) were generated and verified by PCR and RT-qPCR analysis (Supplementary Figures S1, S3). The two complementation strains also exhibited normal morphology as wild type (Supplementary Figure S2), thus a complementation strain (△*FoCupin1*-com) based on △*FoCupin1*-15 was randomly selected for further analyses.

The knockout mutant △*FoCupin1* showed no differences in colony morphology and the growth rate on different medium compared to the wild type and the complementation strain (Figures 4B,G; Supplementary Figure S2). Microscopic examination revealed that the hyphae and conidia morphology of △*FoCupin1* were similar to the wild type (Supplementary Figure S4). To determine whether *FoCupin1* influenced aerial growth, we inoculated these strains into PDB medium, where no difference in dry weight was found between the wild type and the △*FoCupin1* strain (Figure 4H). The △*FoCupin1* strain was also not distinct from the wild type in conidiation in liquid CD medium (Figure 4I). The complementation strain exhibited normal colony morphology, growth rate, and conidiation as the wild type (Figure 4; Supplementary Figure S2). Therefore, our results indicate that FoCupin1 is not required for fungal growth and development.

### *FoCupin1* contributes to Foc TR4 virulence

**Figure 5:**
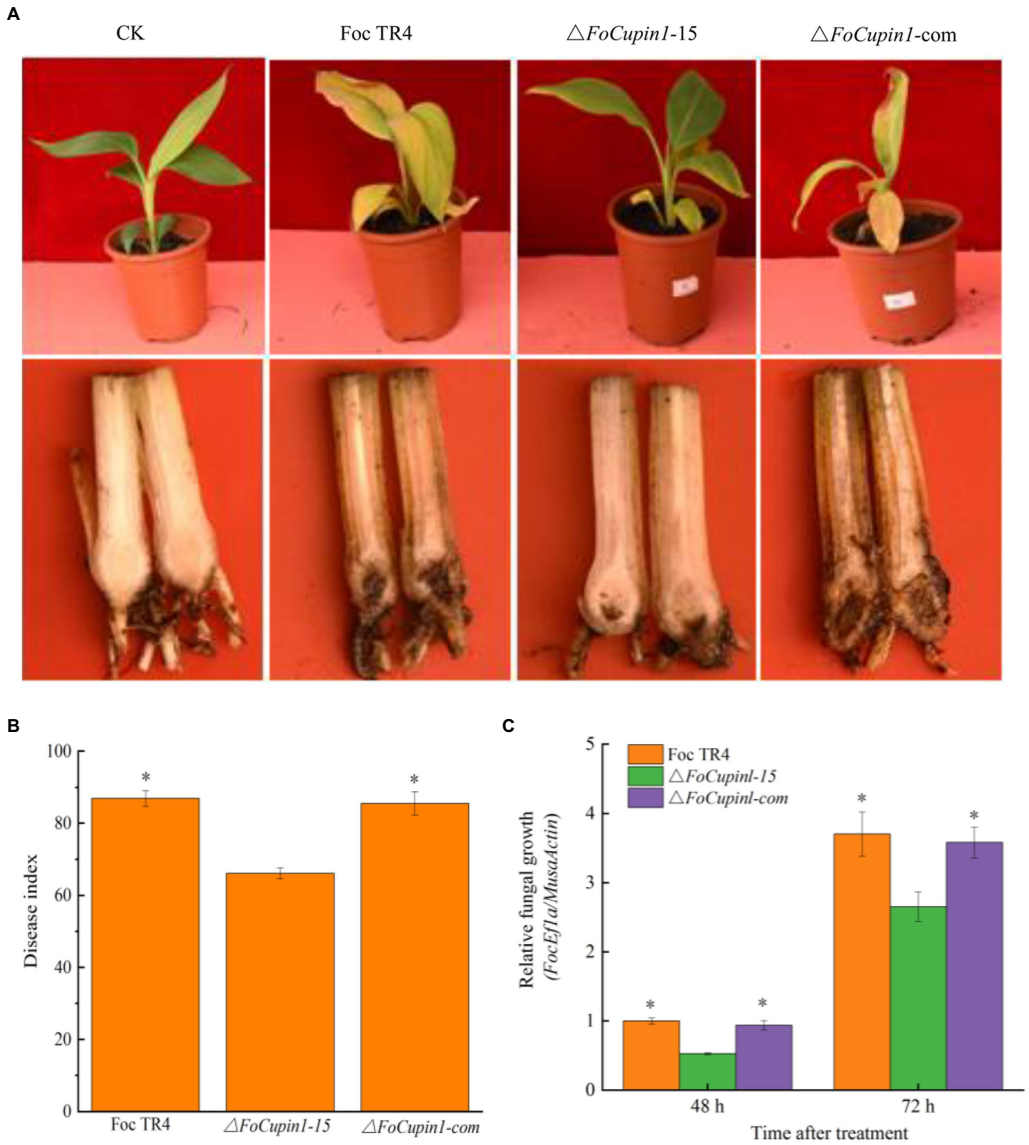
Pathogenicity assay of *FoCupin1* deletion mutant and complementation strain. **(A)** Disease phenotype. Banana plantlets at fully fourth-leaf stage were inoculated with *FoCupin1* deletion mutant and complementation strain. The plantlets were photographed 28 days post-inoculation. **(B)** Disease index. **(C)** Relative biomass assay. The relative fungal growth was measured by [2^[CT(*MaActin*)-CT(*FoEF1a*)]^ × 100] using qPCR. Values are the means (±SE) based on three independent experiments and bars indicate standard deviations. Asterisks indicate statistical significance (*p* < 0.05) using Student’s *t*-test.

In our infection assays with banana roots, the *FoCupin* deletion mutant caused reduced discoloration of the pseudostem, vascular tissue and leaves compared with the wild type and complementation strain (Figure 5A). Moreover, the disease index of the mutant was significantly lower than that of Foc TR4 and the complementation strain (Figure 5B). To further determine whether the disruption of *FoCupin1* affected the fungal growth in planta, we estimated the relative fungal biomass in the infected roots by DNA-based quantitative PCR (qPCR). The assays showed that the relative fungal biomass was lower in the △*FoCupin1*-infected plants than in the Foc TR4-infected plants (Figure 5C). These results showed that *FoCupin1* deletion leads to reduced pathogenicity of Foc TR4 to banana, indicating that *FoCupin1* plays an essential role in Foc TR4 virulence.

### *FoCupin1* deletion has little effect on sensitivity to cell wall, osmotic, and oxidative stress

The sensitivity of *FoCupin1* deletion mutant to osmotic, oxidative and cell wall integrity stress (including SDS, CFW, CR, NaCl, sorbitol, or H_2_O_2_), was also investigated. There was no significant difference in colony morphology and the radial growth between *FoCupin1* deletion mutant and the wild type in the medium supplemented with these multiple stress (Supplementary Figure S5). Therefore, FoCupin1 is likely dispensable for cell wall integrity, osmotic, and oxidative stress responses.

### FoCupin1 is dispensable for fusaric acid production

Fusaric acid (FA) is an important virulence factor of Foc TR4 on banana plantlets (Liu et al., 2020). As *FoCupin1* is necessary for Foc TR4 virulence, the FA content of *FoCupin1* deletion mutant was determined by culturing the spore in banana extracts-containing NCM medium to mimic the host-pathogen interaction. There was no significant difference in the amounts of FA production between *FoCupin1* deletion mutant and the wild type (Supplementary Figure S6), indicating that *FoCupin1* deletion has no effect on FA biosynthesis.

### *FoCupin1* deletion has no effect on fungal penetration

Cellophane penetration is one of the commonly used tests to evaluate invasive growth and virulence of *F. oxysporum* (Zhao et al., 2017). To test whether the attenuated virulence of the *FoCupin1* deletion mutant was due to a penetration defect, a cellophane penetration experiment was conducted. As shown in Supplementary Figure S7A, △*FoCupin1* can penetrate the cellophane sheet as the wild type. To further examine the role of *FoCupin1* on colonization and invasion, the fruits of apple and tomato were further inoculated with mycelial blocks of wild type, *FoCupin1* deletion mutant, and the complementation strain. The results showed that lesions were not significantly different on fruits inoculated with these strains (Supplementary Figures S7B,C). These results suggest that *FoCupin1* deletion does not affect Foc TR4 penetration and colonization.

### *FoCupin1* suppresses plant immunity responses in banana

**Figure 6:**
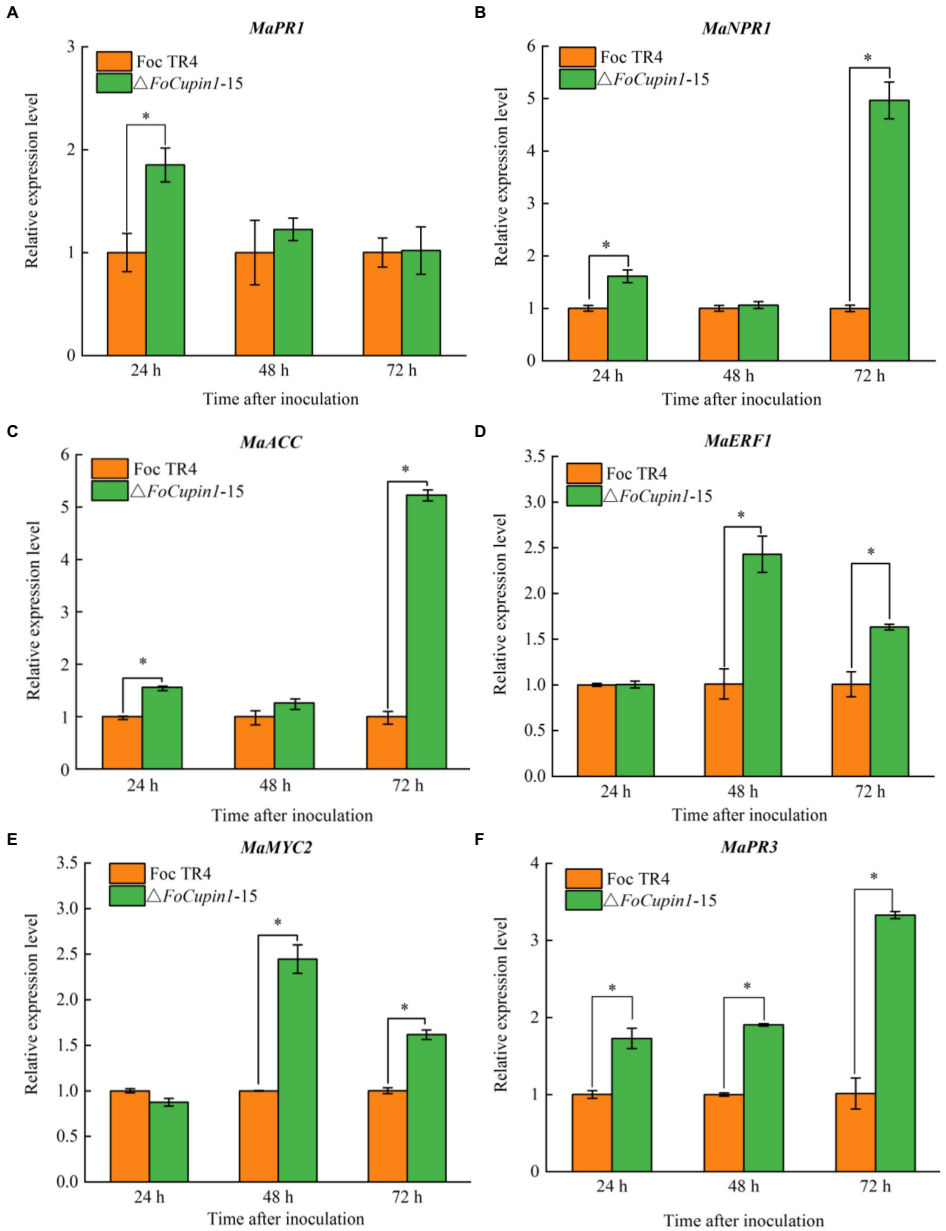
FoCupin1 suppresses the expression of six defense-related genes in banana by RT-qPCR. **(A)** *MaPR1*. **(B)** *MaNPR1*. **(C)** *MaACC*. **(D)** *MaERF1*. **(E)** *MaMYC2*. **(F)** *MaPR3*. Expression levels of these genes were induced stronger during the early stage in △*FoCupin1*-treated banana than wild type-treated bananas. Values are the means (±SE) based on three independent experiments and bars indicate standard deviations. Asterisks indicate statistical significance (*p* < 0.5) using Student’s *t*-test.

To elucidate whether attenuated virulence of △*FoCupin1* was partially due to defect in suppression of plant defense responses, six well-known defense-related genes, namely *MaPR1*, *MaNPR1*, *MaPR3, MaMYC2*, *MaACC*, *MaERF1,* were selected for further RT-qPCR analysis (Booker and Delong, 2015; Qin et al., 2018; Zhang et al., 2019). We compared the expression of genes in banana plants inoculated with △*FoCupin1* or the wild type, and found that: (1) the expression of *MaPR1* was significantly upregulated in banana plants at 24 h after inoculation with △*FoCupin1* (Figure 6A); (2) the expression of *MaNPR1* and *MaACC* was significantly increased at 24 and 72 h (Figures 6B,C); (3) the expression of *MaERF1* and *MaMYC2* was significantly induced at 48 and 72 h (Figures 6D,E); and (4) the expression of *MaNPR3* was significantly upregulated at 24, 48, and 72 h after inoculation (Figure 6F). These results suggest that the deletion of *FoCupin1* may result in the reduced suppression of multiple banana defense responses, which in turn would restrict fungal penetration and proliferation.

### H_2_O_2_ accumulation in banana roots after △*FoCupin1* inoculation

**Figure 7:**
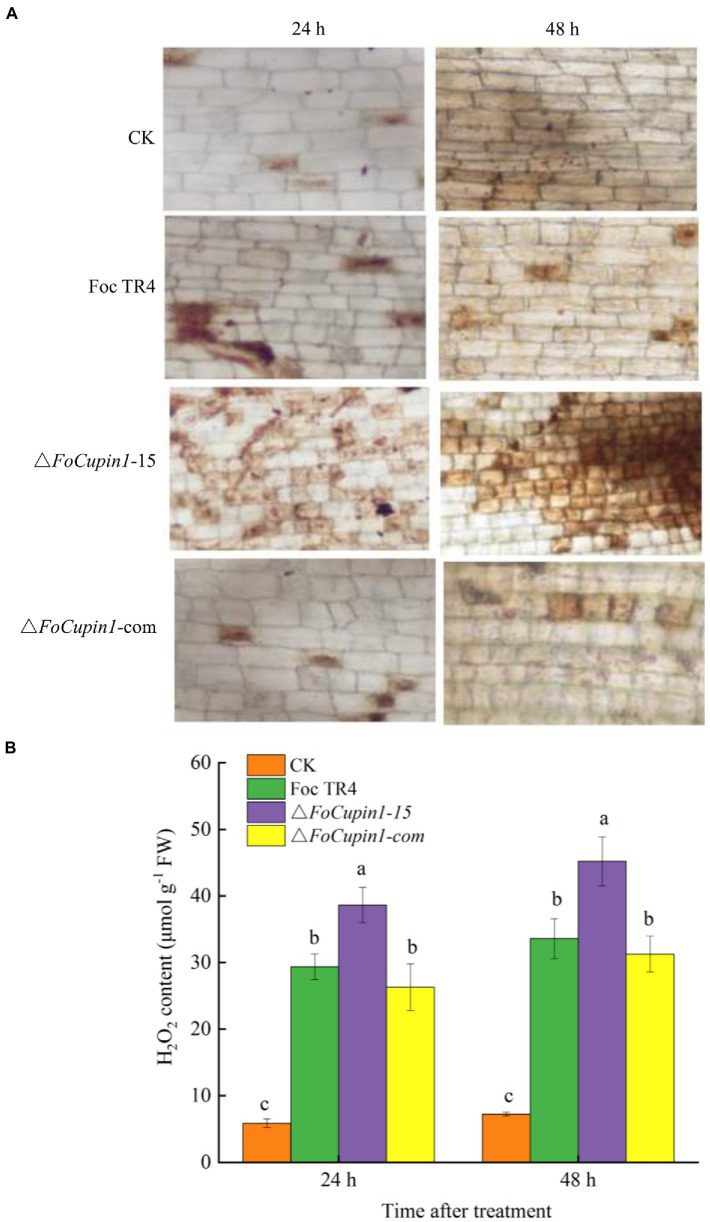
FoCupin1 induces ROS accumulation in banana roots. **(A)** H_2_O_2_ production detected by DAB staining (magnification 100×). **(B)** Quantitative analysis of H_2_O_2_ content in banana roots. Experiments were repeated at least three times. Values are the means (±SE) based on three independent experiments and bars indicate standard deviations. The letters above the bars are significantly different at 0.05 level.

To further investigate whether FoCupin1 is able to suppress plant defense response that is associated with ROS accumulation, we conducted qualitative and quantitative analyses to test the production of H_2_O_2_. As shown in Figure 7A, H_2_O_2_ accumulation was significantly higher in banana roots inoculated with △*FoCupin1* than those inoculated with the wild type or △*FoCupin1*-com. As expected, little staining was observed in the untreated control roots. A study was further conducted to quantify H_2_O_2_ accumulation in banana roots. Consistent with the changes in the histochemical staining results, △*FoCupin1*-treated plants produced significantly higher amounts of H_2_O_2_ than wild type- and △*FoCupin1*-com-treated plants (Figure 7B). These results suggest that *FoCupin1* deletion is not able to suppress ROS accumulation in banana, which further provide enhanced plant defense against pathogen infections.

## Discussion

Banana is the fourth most important fruit crop after wheat, rice and corn, and a major export commodity in much of the developing world. However, banana is continuously threatened by various pathogenic microbes. Fusarium wilt disease caused by Foc4 is the most economically pressing fungal disease of banana, which is now widely distributed in China and has a devastating effect on banana yield (Li et al., 2017). A lack of knowledge of Foc4 pathogenicity hinders the efforts to develop effective methods to control it. Plant pathogens employ effectors as molecular weapons to manipulate host immunity and facilitate colonization (Bi and Zhou, 2021). Recent studies have shown that the effector proteins secreted by Foc4 can act as pathogenicity factors and play important roles in Foc-banana interaction (Li et al., 2019; He et al., 2021). Almost 1,000 putative secreted proteins have been predicted in Foc genome, many of which are thought to be candidates for effector proteins (Guo et al., 2015). A recent study has identified 919 non-redundant secreted proteins in Foc TR4 using label-free quantitative proteomics approach, from which 74 candidate effectors were predicted (Zhao et al., 2020). Expression profiling analyses showed that many putative effectors exhibit enhanced expression during banana infection, indicating that effectors may play significant roles in the interaction between banana and the pathogen (He et al., 2021). However, although Foc4 encodes many putative effector proteins, only a few of them have been studied (Czislowski et al., 2018; An et al., 2019; Chang et al., 2019; Zhao et al., 2020; Zhang et al., 2021; Guo et al., 2022). Here, we characterized a novel new secreted protein FoCupin1 in Foc TR4. Gene expression analysis showed that *FoCupin1* expression increased significantly at the early stage of infection (Figure 2), suggesting that FoCupin1 is likely involved in Foc TR4 pathogenesis.

In this study, *Agrobacterium*-mediated transient expression assay was used to investigate the potential function of FoCupin1 in elicting or suppressing cell death in the non-host *N. benthamiana* plants. We found that FoCupin1 inhibited BAX-mediated cell death and ROS reaction, but could not elicit cell death in *N. benthamiana* leaves (Figure 3). Furthermore, the transient expression of FoCupin1 significantly decreased the expression of four defense marker genes (*NbPR5*, *NbPR4*, *NbLOX*, and *NbEIN2*) in *N. benthamiana* leaves. Cell death induced by the effectors is usually considered as a strategy to facilitate the invasion of necrotrophic pathogens (Lorang et al., 2012). Pathogens often employ effectors to inhibit PTI during compatible interactions, thus enhancing pathogenesis (Fan et al., 2021). Therefore, our results suggest that FoCupin1 may facilitate fungal invasion by suppressing plant defense responses. Similar results have been reported for another putative effector FocM35_1 in Foc TR4, which is able to suppress BAX-induced cell death in *N. benthamiana* (Zhang et al., 2021). We also found that FoCupin1 proteins with or without the signal peptide can both suppress BAX-induced cell death, suggesting that the signal peptide of FoCupin1 was not required for its cell death-suppressing ability in *N. benthamiana*. To better understand the underlying mechanism of FoCupin1 in suppression of the BAX-mediated cell death, future studies should be performed to investigate where this effector protein was translocated into host cells after secretion and, if so, how this effector was recognized by cytoplasmic receptors to trigger cell death.

Cupin proteins represent one of the largest protein superfamilies, which share a double-stranded β-helix fold (Zhang, 2020). Generally, proteins in the cupin superfamily contain one or two cupin structural domains (Shutov and Kakhovskaia, 2011). Cupin proteins possess remarkable functional diversity, as they function as dioxygenase, lyase, epimerase, isomerases, and nonenzymatic storage proteins (Uberto and Moomaw, 2013; Rahimi-Midani et al., 2021; Peng et al., 2022). The cupin superfamily is classified into at least 69 families in the Pfam database, including the Cupin_1 family to which FoCupin1 belongs (Mistry et al., 2021). Previous studies also reported that some proteins from other cupin families contain signal peptides and potentially function as effectors (Abdelbasset et al., 2015; Pei et al., 2019; Fan et al., 2021), indicating a possible important role of cupin superfamily proteins in fungal pathogenesis. However, the biological functions of the Cupin type-1 domain-containing protein remain largely unknown. In this study, through Pfam analysis and BLAST searches against the NCBI database, we identified FoCupin1 which contains a Cupin_1 domain, a N-terminal signal peptide, no transmembrane domain or no GPI-anchor site, and is conserved among many phytopathogenic fungi (Figure 1). Our results also showed that the FoCupin1 protein can function as a putative effector to suppresses plant immunity responses, induce ROS accumulation, and promote the pathogen infection. These findings represents the first example of the functional characterization of a protein in the cupin family.

Several effector proteins that are important for infection have been reported in Foc4. Nine SIX (secreted in xylem) proteins have been identified in Foc4, all of which were significantly induced after inoculation to banana plants (An et al., 2019). Virulence analysis of the SIX2 and SIX8 knock-out mutants showed that SIX8 is required for the virulence of Foc4 while SIX2 has no obvious functions. A secreted metalloprotease, FocM35_1, was found to be an essential virulence effector of Foc TR4 and can inhibit the host immunity (Zhang et al., 2021). Fosp9, a novel secreted effector, was found to be required for the full virulence of Foc4 for banana (Guo et al., 2022). The putative effector encoded by Foc 1,324 gene showed a significant transcriptional burst in planta compared with in-culture conditions, and was required for the pathogenicity of Foc4 (Chang et al., 2019). In this study, FoCupin1 was highly upregulated during the early stages of Foc TR4 infection progress in bananas. The FoCupin1 deletion mutant showed compromised virulence. Importantly, treatment of banana leaves with FoCupin1 deletion mutant induced typical ROS production, and enhanced plant defense against Foc TR4. Therefore, FoCupin1, a novel secreted effector, likely contributes to pathogen virulence by inhibiting the host immunity.

## Data availability statement

The datasets presented in this study can be found in online repositories. The names of the repository/repositories and accession number(s) can be found in the article/Supplementary material.

## Author contributions

TY, XZ, HL, YN, and YL conceived, wrote, reviewed, edited the manuscript and designed the experiments. TY, JL, GL, HW, and YN performed the experiments. TY, XZ, YN, and YL analyzed the data. TY, JL, HL, and YN contributed reagents, materials, and analysis tools. All authors contributed to the article and approved the submitted version.

## Funding

This research was funded by China Agriculture Research System of MOF and MARA (CARS-31), Natural Science Foundation of Guangdong Province (2021A1515010643), the National Natural Science Foundation of China (31600663), Project for Key Technology R&D Innovation Team in Modern Agriculture, Guangdong Province (2021KJ134), and Guangzhou Science and Technology Program (202206010027).

## Conflict of interest

The authors declare that the research was conducted in the absence of any commercial or financial relationships that could be construed as a potential conflict of interest.

## Publisher’s note

All claims expressed in this article are solely those of the authors and do not necessarily represent those of their affiliated organizations, or those of the publisher, the editors and the reviewers. Any product that may be evaluated in this article, or claim that may be made by its manufacturer, is not guaranteed or endorsed by the publisher.

## Footnotes

^1^ http://effectorp.csiro.au/

^2^ http://pfam.xfam.org

^3^ http://www.ncbi.nlm.nih.gov
