# Supplementary material for: FoCupin1, a Cupin_1 domain-containing protein, is necessary for the virulence of Fusarium oxysporum f. sp. cubense tropical race 4
Source: Front Microbiol. 2022 Aug 30;13:1001540. doi: 10.3389/fmicb.2022.1001540 (PMC9468701; doi:10.3389/fmicb.2022.1001540)
Supplement: Supplementary file 1 [file Data_Sheet_1.ZIP › R1_Supplementary materials/R1_Supplementary Materials 1_Figures.pptx]

## Slide 1
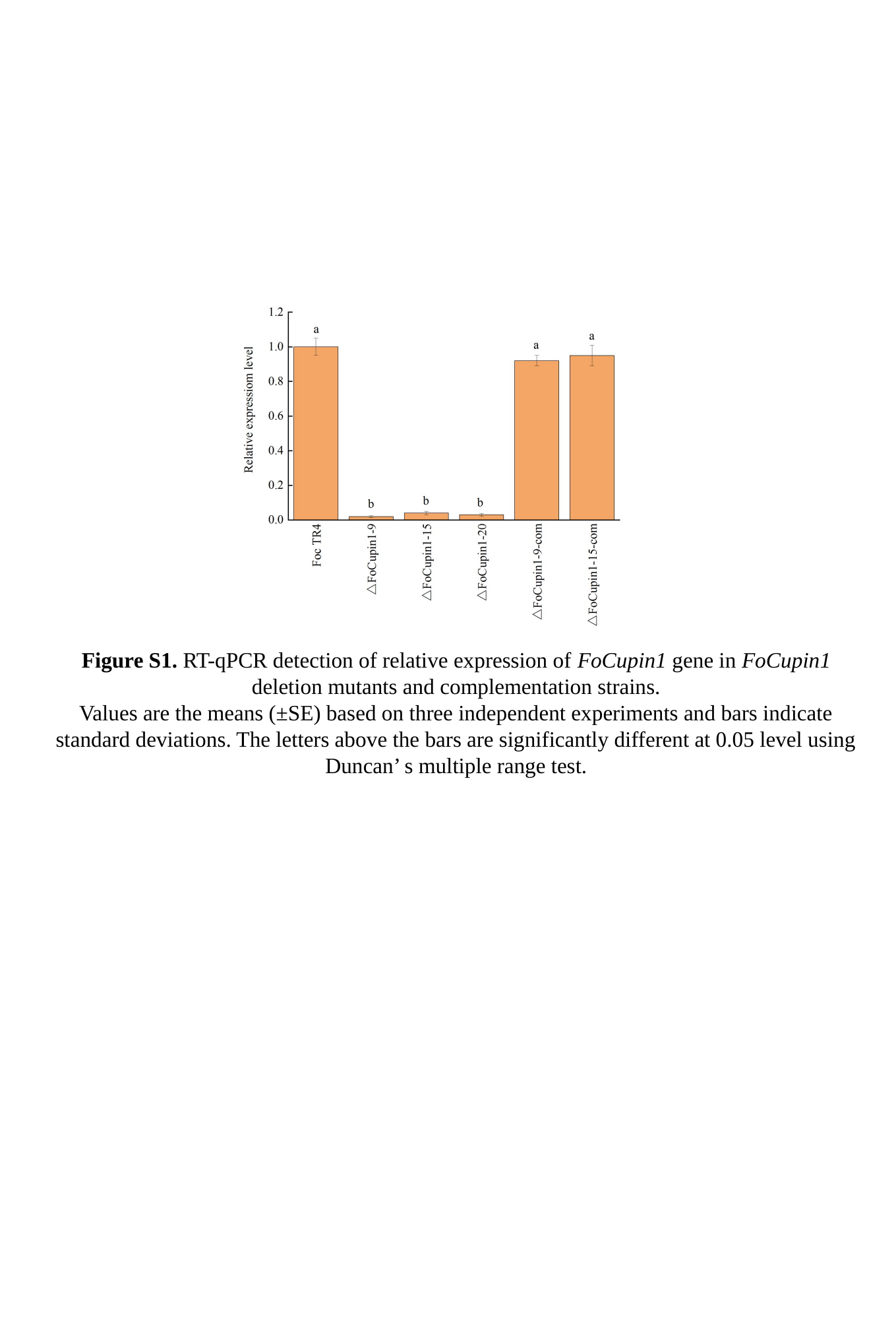

Figure S1. RT-qPCR detection of relative expression of FoCupin1 gene in FoCupin1 deletion mutants and complementation strains.
Values are the means (±SE) based on three independent experiments and bars indicate standard deviations. The letters above the bars are significantly different at 0.05 level using Duncan’ s multiple range test.

## Slide 2
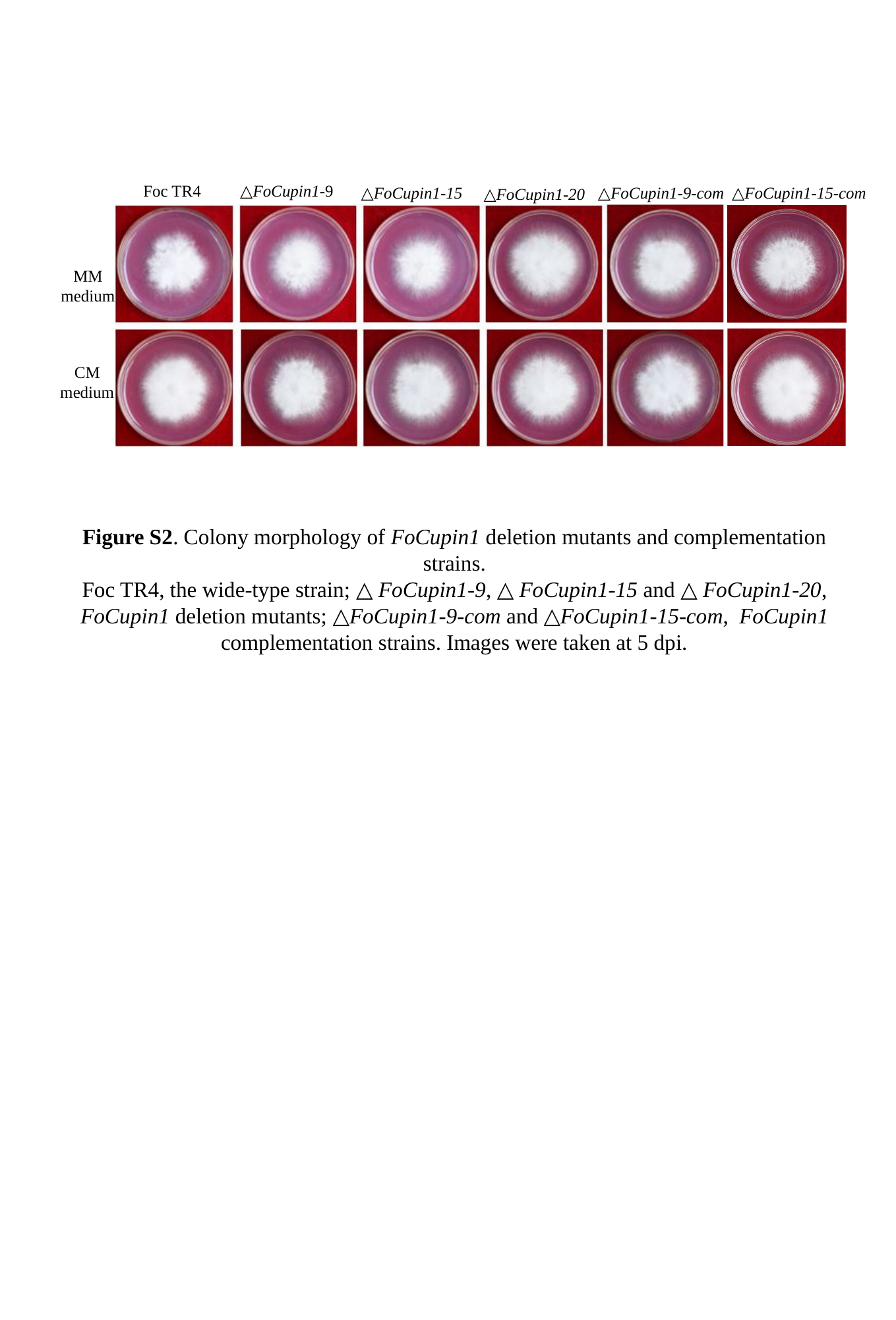

△FoCupin1-9
Foc TR4
△FoCupin1-15
△FoCupin1-9-com
△FoCupin1-15-com
△FoCupin1-20
MM medium
CM
medium
Figure S2. Colony morphology of FoCupin1 deletion mutants and complementation strains.
Foc TR4, the wide-type strain; △ FoCupin1-9, △ FoCupin1-15 and △ FoCupin1-20, FoCupin1 deletion mutants; △FoCupin1-9-com and △FoCupin1-15-com, FoCupin1 complementation strains. Images were taken at 5 dpi.

## Slide 3
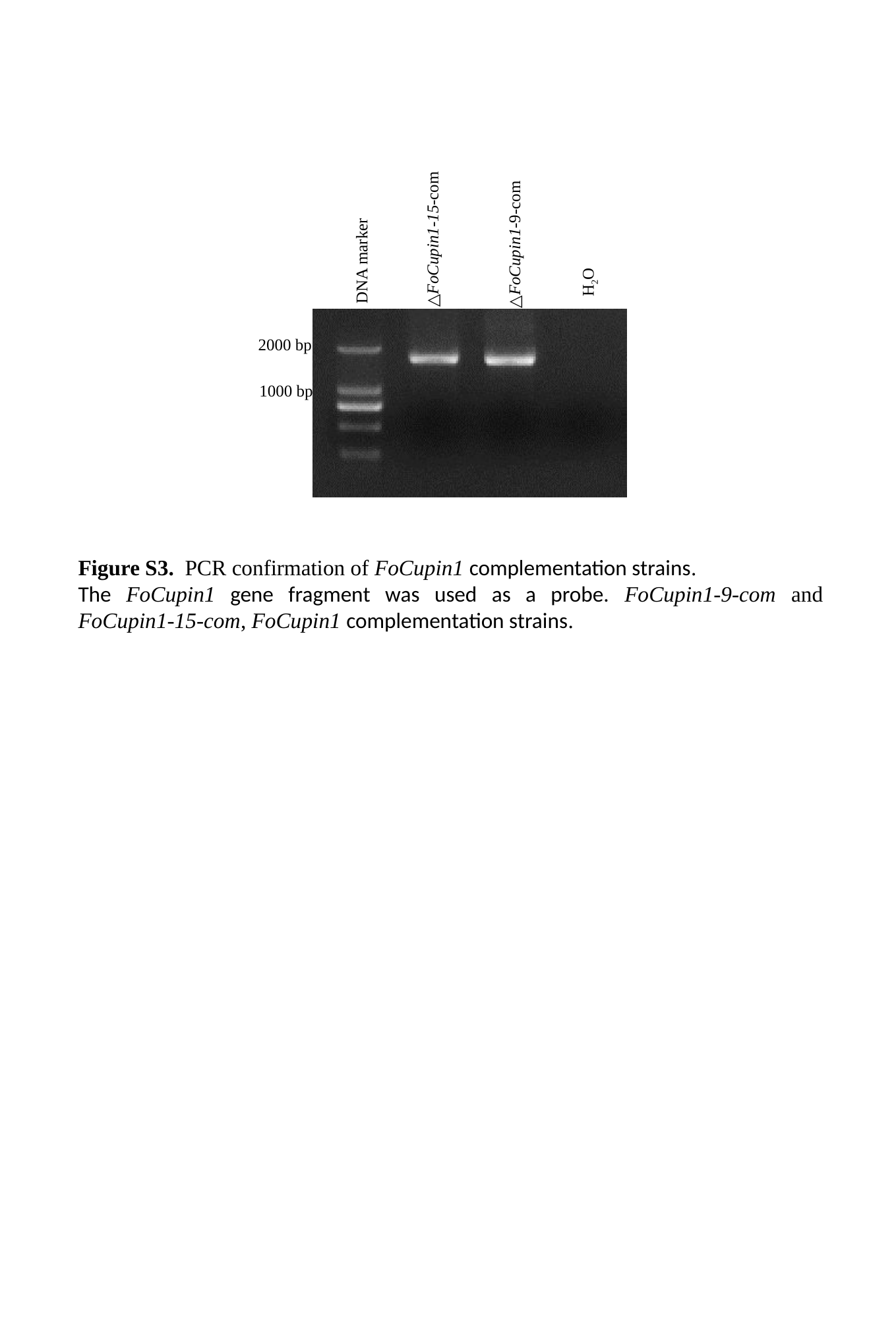

△FoCupin1-9-com
△FoCupin1-15-com
DNA marker
H2O
2000 bp
1000 bp
Figure S3. PCR confirmation of FoCupin1 complementation strains.
The FoCupin1 gene fragment was used as a probe. FoCupin1-9-com and FoCupin1-15-com, FoCupin1 complementation strains.

## Slide 4
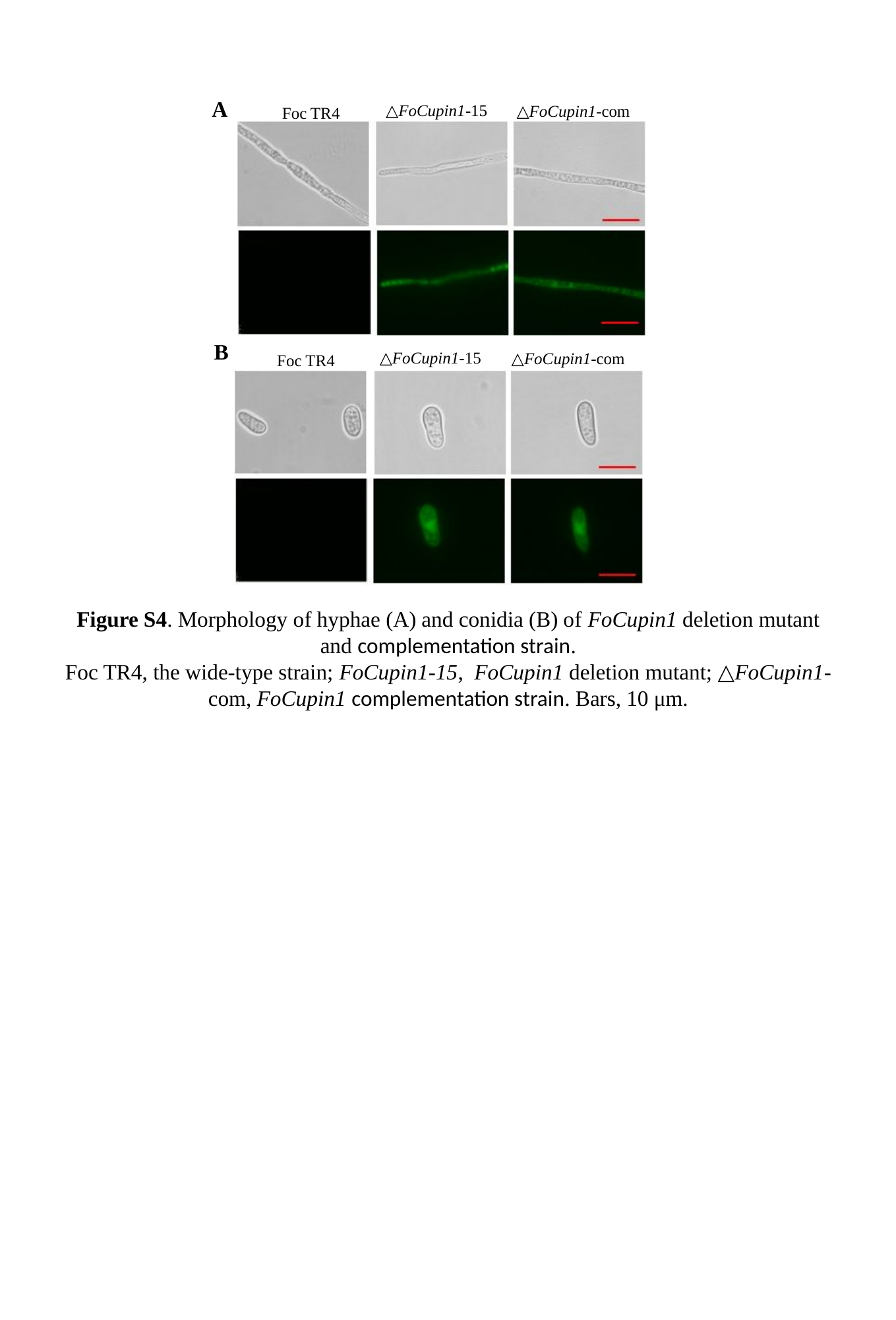

A
△FoCupin1-15
△FoCupin1-com
Foc TR4
B
△FoCupin1-15
△FoCupin1-com
Foc TR4
Figure S4. Morphology of hyphae (A) and conidia (B) of FoCupin1 deletion mutant and complementation strain.
Foc TR4, the wide-type strain; FoCupin1-15, FoCupin1 deletion mutant; △FoCupin1-com, FoCupin1 complementation strain. Bars, 10 μm.

## Slide 5
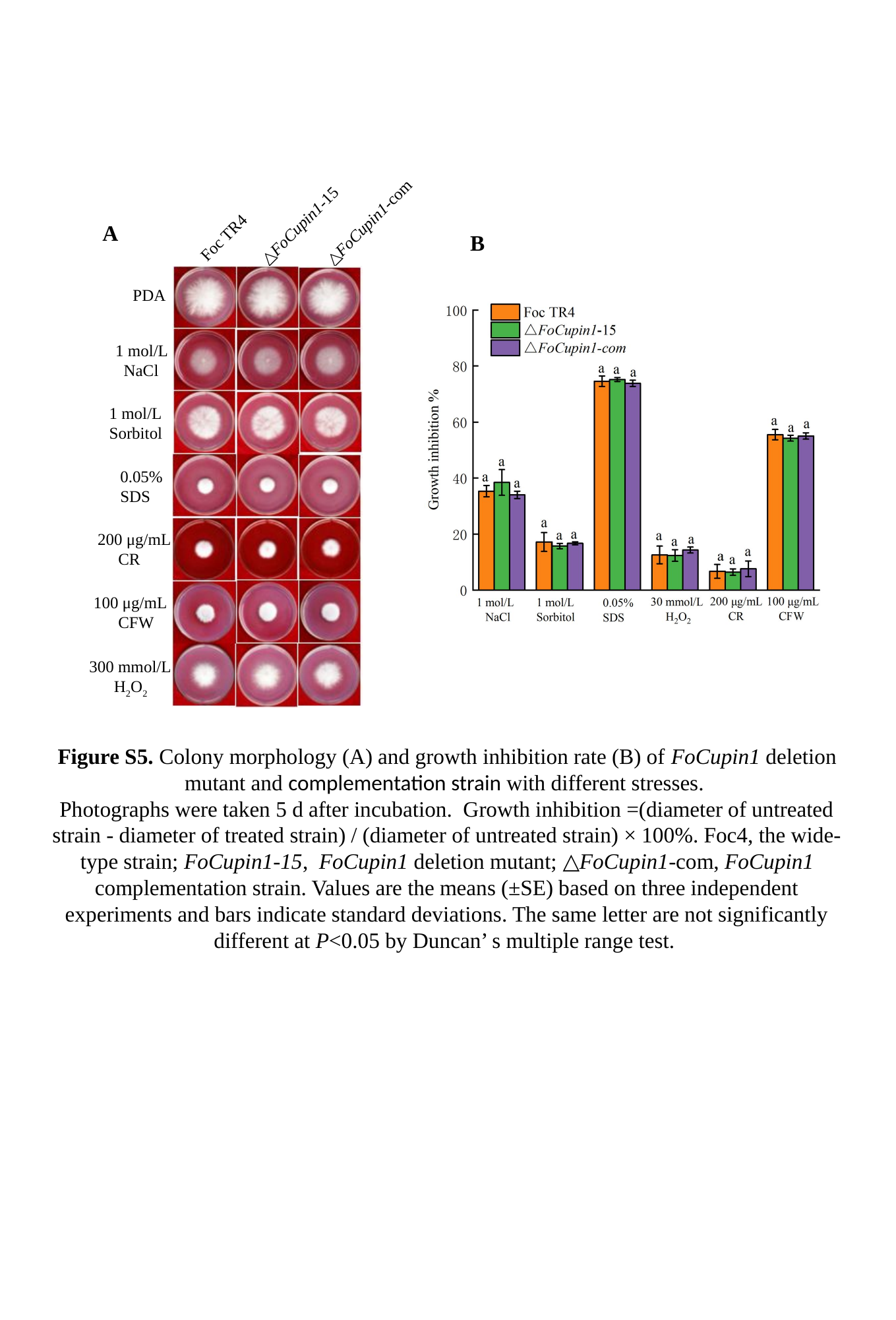

△FoCupin1-com
△FoCupin1-15
A
Foc TR4
PDA
1 mol/L
 NaCl
1 mol/L Sorbitol
0.05% SDS
200 μg/mL
 CR
100 μg/mL
 CFW
300 mmol/L
 H2O2
B
Figure S5. Colony morphology (A) and growth inhibition rate (B) of FoCupin1 deletion mutant and complementation strain with different stresses.
Photographs were taken 5 d after incubation. Growth inhibition =(diameter of untreated strain - diameter of treated strain) / (diameter of untreated strain) × 100%. Foc4, the wide-type strain; FoCupin1-15, FoCupin1 deletion mutant; △FoCupin1-com, FoCupin1 complementation strain. Values are the means (±SE) based on three independent experiments and bars indicate standard deviations. The same letter are not significantly different at P<0.05 by Duncan’ s multiple range test.

## Slide 6
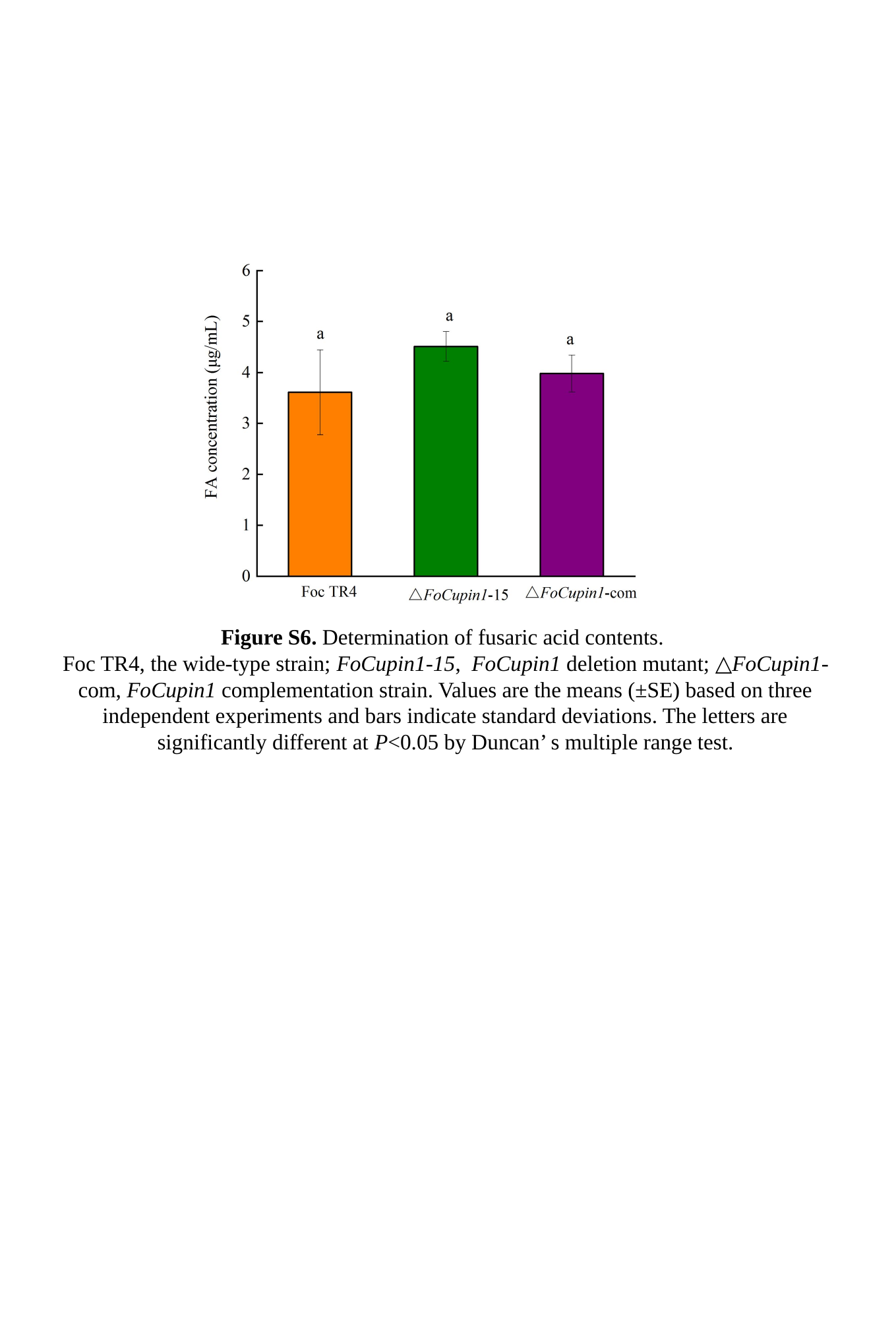

Figure S6. Determination of fusaric acid contents.
Foc TR4, the wide-type strain; FoCupin1-15, FoCupin1 deletion mutant; △FoCupin1-com, FoCupin1 complementation strain. Values are the means (±SE) based on three independent experiments and bars indicate standard deviations. The letters are significantly different at P<0.05 by Duncan’ s multiple range test.

## Slide 7
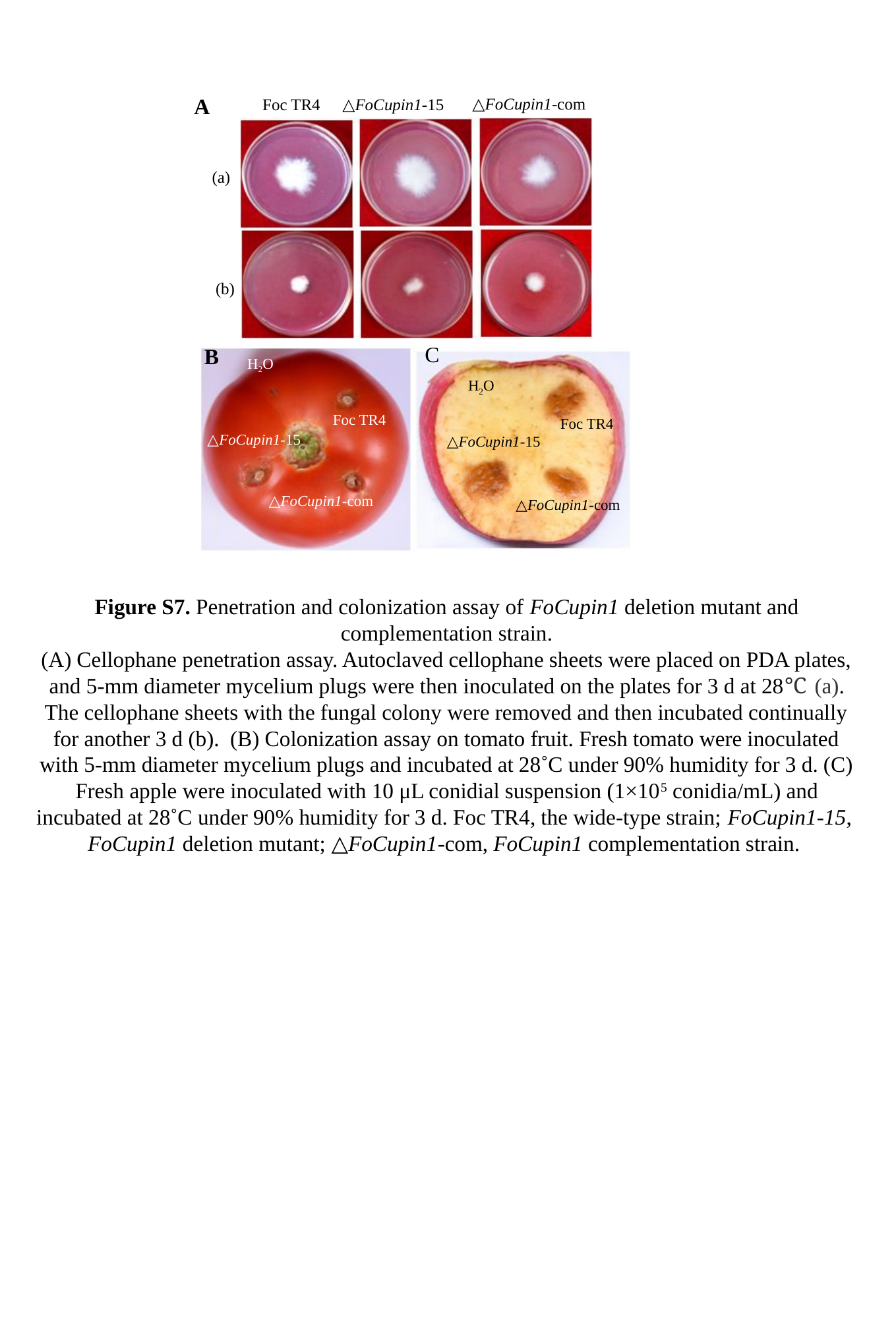

A
△FoCupin1-com
△FoCupin1-15
Foc TR4
(a)
(b)
C
B
H2O
H2O
Foc TR4
Foc TR4
△FoCupin1-15
△FoCupin1-com
△FoCupin1-15
△FoCupin1-com
Figure S7. Penetration and colonization assay of FoCupin1 deletion mutant and complementation strain.
 Cellophane penetration assay. Autoclaved cellophane sheets were placed on PDA plates, and 5-mm diameter mycelium plugs were then inoculated on the plates for 3 d at 28℃ (a). The cellophane sheets with the fungal colony were removed and then incubated continually for another 3 d (b). (B) Colonization assay on tomato fruit. Fresh tomato were inoculated with 5-mm diameter mycelium plugs and incubated at 28˚C under 90% humidity for 3 d. (C) Fresh apple were inoculated with 10 μL conidial suspension (1×10^5^ conidia/mL) and incubated at 28˚C under 90% humidity for 3 d. Foc TR4, the wide-type strain; FoCupin1-15, FoCupin1 deletion mutant; △FoCupin1-com, FoCupin1 complementation strain.
