# Supplementary material for: FoCupin1, a Cupin_1 domain-containing protein, is necessary for the virulence of Fusarium oxysporum f. sp. cubense tropical race 4
Source: Front Microbiol. 2022 Aug 30;13:1001540. doi: 10.3389/fmicb.2022.1001540 (PMC9468701; doi:10.3389/fmicb.2022.1001540)
Supplement: Supplementary file 1 [file Data_Sheet_1.ZIP › R1_Supplementary materials/R1_Supplementary Materials 2_Table S1.docx]

**Table S1.** Primers used in this study

| **Primer name** | **Primer Sequence (5'-3')** | **Reference** |
| --- | --- | --- |
| Construction and confirmation of *FoCupin1* deletion and complementation mutants | | |
| *FoCupin1*-upF | GGACTAGTGCTGCTCAACAACTTCAAATCG | Present work |
| *FoCupin1*-upR | CGGAATTCACATACATCGTGATACAAAGCTTCC | Present work |
| *FoCupin1*-downF | CCGCTCGAGAGCACAGCCGATCTGAAGATGTTT | Present work |
| *FoCupin1*-downR | GGGGTACCCGCGTTTCGAATATCGGTGTG | Present work |
| *hph*-F | TGCTGCTCCATACAAGCCAA | Present work |
| *hph*-R | GACATTGGGGAGTTCAGCGA | Present work |
| *FoCupin1*-F | ACAGATTGCCGGATCCTATCA | Present work |
| *FoCupin1*-R | TGGCCCATAATAGAGGCGGA | Present work |
| *FoCupin1*-comF | GGACTAGTTTCAAACAGTCCCCAGACGG | Present work |
| *FoCupin1*-comR | AAGGAAAAAAGCGGCCGCCACGGGTGCCACGTTACTAT | Present work |
| *FoCupin1* probe-F | GCTCAGTGCGGATAACACGA | Present work |
| *FoCupin1* probe-R | AGTTCACCACTGCTCTCGTC | Present work |
| *hph*-porobe -F | TGCTGCTCCATACAAGCCAA | Present work |
| *hph*-porobe -R | GACATTGGGGAGTTCAGCGA | Present work |
|  | | |
| Construction of transient expression vector | |  |
| SP*FoCupin1*-F | GGACTCTAGAGGATCCATGTACCCATACGATGTTCCAGATTACGCTGGCATGAAGTTCACCACTGCTCTC | Present work |
| SP*FoCupin1*-R | GATCGGGGAAATTCGAGCTC TTAGATGGATCGCTTCTTAAGGC | Present work |
| NSP*FoCupin1*-F | GGACTCTAGAGGATCCATGTACCCATACGATGTTCCAGATTACGCTGGCGCTCCTCGCAACACTCGAC | Present work |
| NSP*FoCupin1*-R | GATCGGGGAAATTCGAGCTCTT AGATGGATCGCTTCTTAAGGC | Present work |
|  | | |
| RT-qPCR analysis for *FoCupin1* in Foc TR4 | | |
| q*FoCupin1*-F | AAGAGCAGGGAAGTTGGCAC | Present work |
| q*FoCupin1*-R | CACTCGACGAACAGACTCCA | Present work |
| q*FoEF1α-*F | GCTGGTGACTCCAAGAACGA | Liu et al., 2019 |
| q*FoEF1α-*R | CATCTTGACGATGGCGGAGT | Liu et al., 2019 |
|  |  |  |
| DNA-based qPCR analysis of fungal biomass | | |
| q*FoEF1α-*F | GCTGGTGACTCCAAGAACGA | Liu et al., 2019 |
| q*FoEF1α-*R | CATCTTGACGATGGCGGAGT | Liu et al., 2019 |
| q*MaActin*-F | TGTTGCATCCTGGTACTGCT | Liu et al., 2019 |
| q*MaActin*-R | GGCTTTCTTGCACTGGTACAC | Liu et al., 2019 |
|  |  |  |
| RT-qPCR analysis of defense related genes in tobacco and banana | |  |
| q*NbPR5*-F | GGGCCAATCTTGGAGCATTA | Seo et al., 2010 |
| q*NbPR5*-R | CAGTCTCCAGTCTCACAATTACC | Seo et al., 2010 |
| q*NbPR4*-F | GGCCAAGATTCCTGTGGTAGAT | Zhang et al., 2017 |
| q*NbPR4*-R | CACTGTTGTTTGAGTTCCTGTTCCT | Zhang et al., 2017 |
| q*NbLOX*-F | AAAACCTATGCCTCAAGAAC | Zhang et al., 2017 |
| q*NbLOX*-R | ACTGCTGCATAGGCTTTGG | Zhang et al., 2017 |
| q*NbEIN2*-F | CGTCAACTATGCTGAACCATTTG | Present work |
| q*Nb EIN2*-R | ACGGGCTGCATGGAATTAT | Present work |
| q*MaPR1-*F | AGGACAACGAGGGGGAGATA | Niu et al., 2018 |
| q*MaPR1-*R | TACGGGTAGGCTGATGGGTT | Niu et al., 2018 |
| q*MaNPR1-*F | GGAGATCCACAAGTAGGTGAAGC | Dalio et al., 2020 |
| q*MaNPR1-*R | AGTCTTGCCAGAGCAACTCG | Dalio et al., 2020 |
| q*MaERF1-*F | CCCAAATGTTGGTCCGTTTC | Dalio et al., 2020 |
| q*MaERF1-*R | TCGCTGTCTTCCACGATTCA | Dalio et al., 2020 |
| q*MaACC-*F | GATGCTGCACATCGGCTAGT | Dalio et al., 2020 |
| q*MaACC-*R | GCCACCTGAATACGGCAGAC | Dalio et al., 2020 |
| q*MaMYC2-*F | CGGATCTACCGACGTGGTCT | Dalio et al., 2020 |
| q*MaMYC2-*R | AGCGTCCGGAGAGCTAAAGT | Dalio et al., 2020 |
| q*NbEF1α*-F | GGTTAAGATGATGCCGACCAAG | Zhang et al., 2017 |
| q*NbEF1α*-R | CGCCAGTTGGGTCCTTCTTG | Zhang et al., 2017 |
| q*MaActin*-F | TGTTGCATCCTGGTACTGCT | Liu et al., 2019 |
| q*MaActin*-R | GGCTTTCTTGCACTGGTACAC | Liu et al., 2019 |
| q*FoEF1α-*F | GCTGGTGACTCCAAGAACGA | Liu et al., 2019 |
| q*FoEF1α-*R | CATCTTGACGATGGCGGAGT | Liu et al., 2019 |

**References in supplementary materials**

Dalio, R. J. D., Maximo, H. J., Roma-Almeida, R., Barretta, J. N., José, E. M., Vitti, A. J., et al. (2020). Tea tree oil induces systemic resistance against Fusarium wilt in bBanana and Xanthomonas infection in tomato plants. *Plants (Basel)* 9(9):1137. doi: 10.3390/plants9091137.

Liu, S., Wu, B., Yang, J., Bi, F., Dong, T., Yang, Q., et al. (2019). A cerato-platanin family protein FocCP1 is essential for the penetration and virulence of *Fusarium oxysporum* f. sp. *cubense* tropical race 4. *Int. J. Mol. Sci.* 20(15):3785. doi: 10.3390/ijms20153785.

Niu, Y., Hu, B., Li, X., Chen, H., Takáč, T., Šamaj, J., et al. (2018). Comparative digital gene expression analysis of tissue-cultured plantlets of highly resistant and susceptible banana cultivarsin response to *Fusarium oxysporum*. *Int. J. Mol. Sci.* 19(2):350. doi: 10.3390/ijms19020350.

Seo, P. J., Kim, M. J., Park, J. Y., Kim, S. Y., Jeon, J., Lee, Y. H., et al. (2010). Cold activation of a plasma membrane-tethered NAC transcription factor induces a pathogen resistance response in *Arabidopsis*. *Plant J.* 61(4):661-71. doi: 10.1111/j.1365-313X.2009.04091.x.

Zhang, L., Ni, H., Du, X., Wang, S., Ma, X. W., Nürnberger, T., et al. (2017). The Verticillium-specific protein VdSCP7 localizes to the plant nucleus and modulates immunity to fungal infections. *New Phytol.* 215(1):368-381. doi: 10.1111/nph.14537.
